# Clusters of Multidrug-Resistant *Mycobacterium tuberculosis* Cases, Europe

**DOI:** 10.3201/eid1507.080994

**Published:** 2009-07

**Authors:** Isabelle Devaux, Kristin Kremer, Herre Heersma, Dick Van Soolingen

**Affiliations:** European Centre for Disease Prevention and Control, Stockholm, Sweden (I. Devaux); National Institute for Public Health and the Environment, Bilthoven, the Netherlands (K. Kremer, H. Heersma, D. Van Soolingen)

**Keywords:** MDR TB, cluster, European surveillance, Beijing genotype, research

## Abstract

Molecular surveillance of multidrug-resistant tuberculosis (MDR TB) was implemented in Europe as case reporting in 2005. For all new MDR TB cases detected from January 2003 through June 2007, countries reported case-based epidemiologic data and DNA fingerprint patterns of MDR TB strains when available. International clusters were detected and analyzed. From 2003 through mid-2007 in Europe, 2,494 cases of MDR TB were reported from 24 European countries. Epidemiologic and molecular data were linked for 593 (39%) cases, and 672 insertion sequence *6110* DNA fingerprint patterns were reported from 19 countries. Of these patterns, 288 (43%) belonged to 18 European clusters; 7 clusters (242/288 cases, 84%) were characterized by strains of the Beijing genotype family, including the largest cluster (175/288 cases, 61%). Both clustering and the Beijing genotype were associated with strains originating in eastern European countries. Molecular cluster detection contributes to identification of transmission profile, risk factors, and control measures.

## Abstract 2

These case investigations may help identify routes of transmission.

Prevalence of multidrug-resistant tuberculosis (MDR TB), i.e., TB resistant to at least rifampin and isoniazid, is increasing, particularly in some areas of Europe (*1*). From 1999 through 2002, the median prevalence of MDR TB in new case-patients was at critical levels (>6.5%) in specific regions of the world, including the Baltic states and other eastern European countries (*2*). In 2004, the Russian Federation, China, and India accounted for 62% of the estimated global MDR TB cases (*3*). The increases in prevalence and incidence of MDR TB are most likely caused by inadequate treatment regimens (*4*). Recently, public health awareness about MDR TB has been reenforced by the occurrence of extensively drug-resistant (XDR) TB outbreaks associated with HIV, particularly in South Africa (*5**,**6*). Surveillance of drug resistance, based on annual case reporting of drug susceptibility tests, has been ongoing in Europe since 1998 and includes annual reporting of MDR TB cases after the start of treatment (*7*). In the 1990s, molecular methods became available and have allowed researchers to study issues in the epidemiology of TB (*8*). Strains of the *Mycobacterium tuberculosis* Beijing genotype, which constitute a group of genetically closely related strains (*9*), have been associated with high levels of drug resistance in countries of the former Soviet Union (FSU), including Latvia (*10*) and Estonia (*11*); these strains have been of low prevalence (6%–7%) in Western Europe (*12*).

Public health officials are concerned about possible emergence and transmission of MDR TB strains across Europe (cross-border migration), but no system is in place to identify whether MDR TB strains are shared (clustered) among European countries. Risk factors for possible clustering have yet to be determined. When the drug resistance surveillance project began, data on MDR TB cases were reported in aggregated format at the European level and could not be linked to molecular data identified from individual MDR TB cases. Also, a need existed to further identify the association between Beijing genotype strains or *M. tuberculosis* strains of other genotypes and MDR TB in Europe.

Molecular surveillance of MDR TB was developed in Europe in recent years, first, in a pilot phase as part of the European Concerted Action on Tuberculosis project and, prospectively, since 2005, with the MDR TB project presented in this paper (*13*). The objectives of this project were to identify molecular clusters of MDR TB cases reported in >1 European country, to describe epidemiologic risk factors associated with MDR TB cases, and to initiate cluster investigations at the national level to prevent cross-border transmission.

In this article, based on surveillance data, we describe the main epidemiologic and demographic characteristics of MDR TB cases reported from January 2003 through June 2007 in 24 European countries. We also describe characteristics of the main European clusters and identify risk factors for clustering and association with the Beijing strain of *M. tuberculosis*.

## Materials and Methods

### Data Collection and Management

In 2005, all 53 countries of the World Health Organization’s European region were invited to participate in the MDR TB surveillance project by Euro TB and the National Institute for Public Health and the Environment (RIVM). Countries were encouraged to provide both epidemiologic and genotyping data on MDR TB cases reported since January 1, 2003. However, countries that could provide only epidemiologic data were also included in the surveillance project. National surveillance institutions sent quarterly updates of individual and anonymous data on MDR TB cases reported since 2003 to EuroTB, according to a standardized data file specification (www.eurotb.org/mdr_tb_surveillance/pdf/DFS_NSI_short.pdf). Each patient had a unique record identifier by country. Common definitions of variables were used by participating countries, including demographic, clinical, and genotyping information. In 18 countries, the country of origin was defined as the country of birth. Because of confidentiality constraints, country of birth was not reported for some countries. Country of citizenship was used to qualify the origin of patients for 2 countries. Either country of birth or country of citizenship was used in 3 countries, and information on geographic origin was unavailable in 1 country. A patient had to be reported again (with a new identifier) when a new MDR *M. tuberculosis* isolate was obtained >24 months after the previously reported MDR TB isolate. However, the possibility of a case being reported twice in the same country or in 2 countries was low.

National laboratories sent insertion sequence (IS) *6110* restriction fragment length polymorphism (RFLP) patterns from available MDR TB strains collected since 2003 to the RIVM every 3 months, either as a Bionumerics bundle (Applied Maths, Sint-Maartens-Latem, Belgium) or as a scanned image (*14*). To be included in the project, laboratories had to perform drug susceptibility testing and participate in an international quality assurance program. For quality assurance, either participating laboratories represented supranational reference laboratories of the World Health Organization and the International Union against Tuberculosis and Lung Disease and participated in the yearly proficiency testing for isoniazid, rifampin, streptomycin, and ethambutol (*15**,**16*), or laboratories had their external quality assurance conducted by such a reference laboratory. For 1 country, drug susceptibility testing was performed in a laboratory abroad. More details (e.g., on drug susceptibility test methods) can be found in the 2006 EuroTB report (*17*).

European cluster information was communicated by the RIVM (Bilthoven, the Netherlands) to EuroTB on a quarterly basis. The final database was maintained by EuroTB and included 3 sections: A) demographic and clinical variables, B) cluster information on the basis of molecular patterns at the country level, and C) European cluster information. Data from sections A and B were matched according to a patient code; data from sections B and C were matched using the strain code attributed at the national level.

### Analysis of Genotyping Data

IS*6110* RFLP was the recommended genotyping method (*18*) to report IS*6110* RFLP patterns to RIVM. For most countries, genotyping was performed locally; for 2 countries, genotyping was performed at RIVM. A European cluster was defined as >2 MDR TB cases with *M. tuberculosis* isolates that shared identical IS*6110* RFLP patterns in >2 countries (*18*). A national cluster included cases diagnosed in a single country. Determination of a national cluster (a cluster including MDR TB cases diagnosed in 1 participating country) could be based on 3 typing methods: IS*6110* RFLP typing (*18*), spoligotyping (*19*), and/or typing that used mycobacterial interspersed repetitive units with variable numbers of tandem repeats (*20*).

In each laboratory, quality control of the molecular typing practices and computer-assisted analysis was included as described in the standardized methods (*14*). The DNA fingerprint patterns of MDR TB strains received at the RIVM were submitted to the molecular database managed by the RIVM (MDRTBase) by using the Bionumerics software (Applied Maths), and the database manager performed a quality check. The IS*6110* RFLP patterns newly added to the MDRTBase were then compared with all other patterns stored in this database. The MDR TB strains were classified as clustered (included in a European cluster) or “unique” (not included in a European cluster). In addition to specifying European cluster reports, the database manager also specified strains belonging to the Beijing genotype family of *M. tuberculosis* by compariing them with the 19 reference RFLP patterns of Beijing strains described by Kremer et al. (*21*).

### Statistical Data Analysis

Factors associated with clustering or infection with a Beijing genotype strain were determined by the unadjusted and adjusted logistic regression model using SAS software (SAS Institute, Cary, NC, USA). The explanatory variables were demographic (sex, age, origin) and clinical (pulmonary vs. extrapulmonary TB, TB history) characteristics of MDR TB cases. XDR TB was studied as an explanatory variable for patients with drug susceptibility results meeting the definition of XDR TB (*22*) in both cluster and strain analyses. The proportion of clustering among Beijing strains was compared with the proportion of clustering among strains of other genotypes.

Because of the limited numbers per category, “unknown” categories were removed from all variables included in the model, as was the age category “<15 years” and the category “other” for the variable country of origin. Analyses, including clustering and the XDR variable, were performed in the univariate model only because of the variables’ strong association with origin in the Baltic states (χ^2^ 97.1, p<0.001) and other countries of the FSU (χ^2^ 20.5, p<0.001).

## Results

### Country Participation, Number of MDR TB Cases Reported, and Clustering of MDR TB Cases

Twenty-four countries from the European Union and western Europe plus Croatia and Macedonia participated in the MDR TB project (Table 1). Epidemiologic data on 2,494 MDR TB cases reported from January 2003 through July 2007 were sent to EuroTB. The Baltic states (Estonia, Latvia, and Lithuania) reported 1,616 cases, representing 65% of total cases. In some countries, epidemiologic data were reported for <3 years (Germany, Lithuania, Poland, Romania, and Spain).

**Table 1:** Number of diagnosed MDR TB cases reported in 24 European countries and number and proportion of genotyped MDR TB strains, as reported to the MDR TB surveillance project, by year, January 2003 through June 2007*

Country	No. cases reported						No. genotyped strains reported	
	2003	2004	2005	2006 and 2007	Total		All	With data (% total cases)
Belgium† (EU)	9	14	7	1	31		15	15 (48)
Croatia	2	1	2	–	5		5	5 (100)
Cyprus (EU)	2	0	1	–	3		–	–
Czech Republic (EU)	15	13	4	6	38		11	5 (13)
Denmark (EU)	0	0	5	–	5		4	4 (80)
Estonia (EU)	96	80	72	–	248		228	228 (92)
Finland (EU)	3	0	2	2	7		7	7 (100)
France (EU)	53	53	46	–	152		63	63 (41)
Germany (EU)	–	–	101	–	101		17	16 (16)
Ireland (EU)	1	2	3	2	8		3	3 (38)
Israel	18	15	12	–	45		39	39 (87)
Italy (EU)	45	18	17	3	83		25	25 (30)
Latvia (EU)	188	208	160	156	712		–	–
Lithuania (EU)	–	318	338	–	656		56	56 (9)
Macedonia, FYR	4	1	6	4	15		–	–
Netherlands (EU)	17	10	7	–	34		44	34 (100)
Norway (EU)	2	4	3	2	11		7	3 (27)
Poland (EU)	–	–	1	16	17		–	–
Romania (EU)	–	25	25	–	50		–	–
Slovenia (EU)	1	0	1	1	3		2	2 (67)
Spain (EU)	–	28	22	–	50		50	50 (100)
Sweden (EU)	7	7	4	3	21		21	18 (86)
Switzerland	9	9	3	4	25		25	20 (80)
United Kingdom (EU)	68	52	54	–	174		50	–
Total	540	858	896	200	2,494		672	593 (39)

*MDR TB, multidrug-resistant tuberculosis; EU, European Union; –, not available; FYR, Former Yugoslav Republic of Macedonia. †Includes only cases detected at the start of treatment.

**Figure 1:**
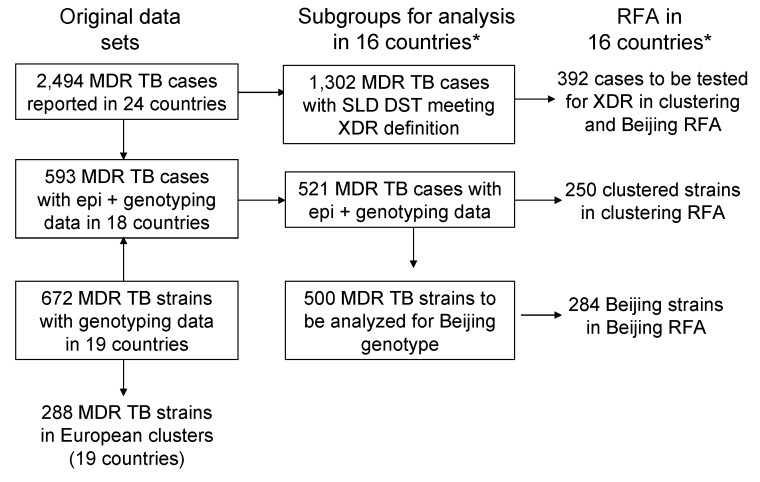
Description of MDR TB cases in Europe selected for data analysis, January 2003–July 2007. RFA does not include data from Germany and Lithuania. MDR TB, multidrug-resistant tuberculosis; SLD, second-line drug; DST, drug-susceptibility test; RFA, risk factor analysis. *Countries: Belgium, Czech Republic, Denmark, Estonia, Finland, France, Germany, Ireland, Israel, Lithuania, the Netherlands, Norway, Spain, Sweden, Switzerland, and the United Kingdom.

Laboratories from 19 countries sent 672 IS*6110* RFLP patterns to the RIVM, 593 of which also had epidemiologic information and were linked to the epidemiologic database maintained by EuroTB (Table 1; Figure 1). Linkage of molecular and epidemiologic data was not possible for data from the United Kingdom and for data on a few cases from 6 other countries. The average proportion of MDR TB cases documented with both epidemiologic and genotyping data in 18 countries was 593/1,523 (39%), varying from 9% to 100%. After we removed data from 2 countries with low data completeness for genotypes (Germany and Lithuania), we found that the average proportion of MDR TB cases with both epidemiologic and genotyping data was 68%. Individual data on second-line drug tests for 2 to 5 second-line drugs included in the XDR TB definition (*22*) were reported for 1,302 cases by 16 countries from January 2003 through July 2007 (Figure 1).

Of the 672 MDR TB strains with IS*6110* RFLP patterns reported, 288 (43%) were identified as belonging to European clusters (Table 2; Figure 1). The proportion of MDR TB strains included in European clusters was <20% in 7 countries (Croatia, France, Italy, Slovenia, Spain, Switzerland, and the United Kingdom). A large proportion (38%) of the RFLP patterns submitted to the molecular database originated from Estonia, where 80% (183/228) of the strains clustered. The number of MDR TB strains identified in national clusters was 170 among 330 strains (51%) reported in 13 of the 19 countries with genotyping data. In these 13 countries, the proportion of intercountry clustering was 28%.

**Table 2:** Number of genotyped MDR TB strains in 19 European countries, with cluster status, by country, January 2003 through June 2007*

Country	Genotyped strains	European clusters, no. (%)	National clusters, no. (%)
Belgium†	15	7 (47)	7 (47)
Croatia	5	0	5 (100)
Czech Republic	11	3 (27)	–
Denmark	4	1 (25)	1 (25)
Estonia	228	183 (80)	–
Finland	7	3 (43)	5 (71)
France	63	10 (*16*)	24 (38)
Germany	17	8 (47)	9 (53)
Ireland	3	1 (33)	–
Israel	39	18 (46)	32 (82)
Italy	25	0	–
Lithuania	56	18 (32)	43 (77)
Netherlands	44	11 (25)	17 (39)
Norway	7	2 (29)	2 (29)
Slovenia	2	0	2 (100)
Spain	50	7 (14)	14 (28)
Sweden	21	9 (43)	9 (43)
Switzerland	25	3 (12)	–
United Kingdom	50	4 (8)	–
Total	672	288 (43)	170 (51)

*MDR TB, multidrug-resistant tuberculosis; –, not available. †Includes only cases detected at the start of treatment.

### Description of MDR TB Clusters

Eighteen distinct European clusters (range 2–175 cases) were identified in 16 countries (Table 3). Clusters E0051, E0054, E0055, E0057, E0063, E0066, and E0069 were characterized by Beijing genotype strains, comprising 242/288 (84%) clustered MDR TB cases. The characteristics of the 4 largest clusters are described below.

**Table 3:** Number of multidrug-resistant tuberculosis strains in 16 European countries, by cluster and by country, January 2003 through June 2007

Country	Cluster no. (E00--)																		Total
	51*	54*	55*	53†	64	68	60	66*	70†	57*	63*	67†	56	59	61	62	65	69*	
Estonia	148	18	13					2			2								183
Israel	8	3	2		2			2					1						18
Lithuania	6	3	1		4		2										1	1	18
Netherlands	1	4	1	2		1									1	1			11
France				6			1		1	1				1					10
United Kingdom	1		1	1								1							4
Sweden	1	2	1						1	1		1		1		1			9
Germany	1	3	2							1	1								8
Belgium	3			2									1		1				7
Spain						5			1									1	7
Switzerland	2			1															3
Czech Republic							2										1		3
Finland	2											1							3
Norway	1		1																2
Denmark									1										1
Ireland	1																		1
Total	175	33	22	12	6	6	5	4	4	3	3	3	2	2	2	2	2	2	288

*Caused by Beijing genotype strains. †Caused by low-copy strains; the insertion sequence i restriction fragment length polymorphism pattern of cluster E0053 consisted of 4 bands; cluster E0070, 3 bands; and E0067, 1 band.

#### Cluster E0051

**Figure 2:**
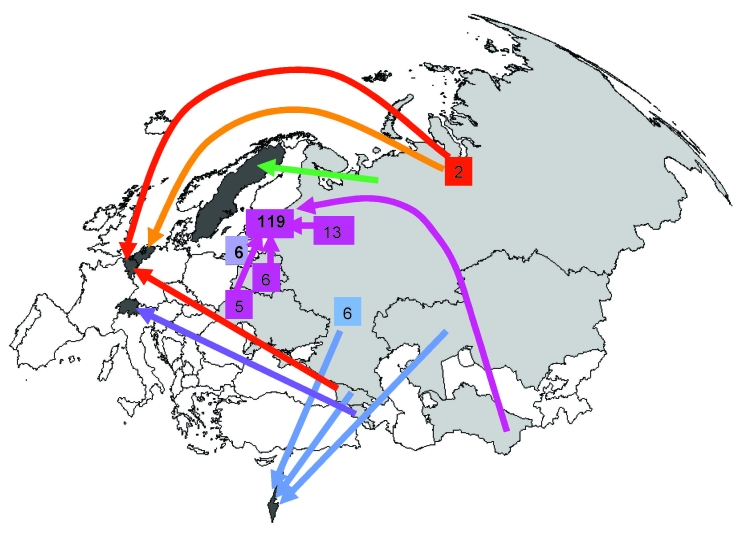
Origin and country of report for the largest European cluster of multidrug-resistant tuberculosis cases (cluster E0051). The cases were reported in 12 European countries (see Table 3). Five countries did not have information about the origin of the cases reported there. For cases reported in the countries shaded in dark gray, the base of the arrow shows where case-patients originated (1 case per arrow, unless otherwise indicated). The 6 case-patients reported in Lithuania were also born there. Of the 148 case-patients reported in Estonia, 119 were also born there. The remaining case-patients reported in Estonia were originally from Russia (13), Belarus (6), Ukraine (5), Lithuania (1) and Turkmenistan (1); origin was unknown for 3 case-patients.

The largest cluster, E0051, consisted of 175 MDR TB cases reported in 12 countries. Of these 175 cases, 148 (85%) were reported in Estonia. No significant differences were found between case-patients included in cluster E0051 and those included in other clusters for sex, age category, TB history, and XDR TB (χ^2^ test, p>0.05). A large proportion (119/148, 81%) of the case-patients reported in Estonia also originated from this country (Figure 2). The proportion of case-patients who had never had TB (either no diagnosis or no treatment) was high (90/148, 61%). Thirty-three cases (22%) were identified as XDR TB. Eight of the 175 case-patients in cluster E0051 were reported in Israel, and all 8 originated from FSU countries (6 from the Russian Federation, 1 from Georgia, and 1 from Kazakhstan); all were pulmonary patients and alive at the time of diagnosis. No information was available on previous TB diagnosis or treatment. None of the 8 MDR TB cases reported in Israel were XDR. The 6 case-patients reported in Lithuania also originated from that country. Five of these case-patients were known to have had a previous diagnosis of TB; XDR TB was not diagnosed in these case-patients. The 3 case-patients reported in Belgium originated from the Russian Federation (n = 2) and from Georgia. Their TB history was unknown. For the 2 case-patients reported in Finland, origin was unknown. One of the 2 case-patients reported in Switzerland was from Armenia; the origin of the other case-patient was unknown. The 2 case-patients reported in the Netherlands and Sweden originated from the Russian Federation.

#### Cluster E0054

Twenty-eight of the 33 case-patients included in the second largest cluster, E0054, had a known origin. All but 1 (from Israel) originated from FSU countries (17 originating and reported in Estonia).

#### Cluster E0055

Eighteen of the 22 case-patients included in cluster E0055 had a known origin; 13 were reported in Estonia, 2 in Israel, 1 in Lithuania, 1 in the Netherlands, and 1 in Sweden. All originated from FSU countries, except the case-patient in the Netherlands, who originated from Sri Lanka.

#### Cluster E0053

Cluster E0053 (non-Beijing) included 10 cases with known origin. Five case-patients originated from the country of report, and 5 others originated from Africa (2 from Côte d’Ivoire, 2 from Nigeria, and 1 from Mali). Because this cluster was characterized by an *M. tuberculosis* strain containing 4 copies of IS*6110*, it could possibly be subdivided if additional DNA typing methods are used. As indicated in Table 3, two other clusters were also caused by low–copy-number strains: clusters E0070 (4 cases) and E0067 (3 cases).

### Risk Factors for Clustering Among MDR TB Isolates

Risk factors for clustering were analyzed for 521 cases documented with both molecular and epidemiologic data reported by 16 countries. Lithuania and Germany were excluded from this analysis due to incomplete genotyping data. Among these 521 cases, 250 (48%) belonged to European clusters and 271 (52%) had isolates with unique DNA fingerprint patterns (Figure 1). The results of univariate and multiple logistic regression analyses to determine factors associated with clustering are presented in Table 4.

**Table 4:** Predictors for clustered MDR TB strains compared with unique strains among 521 cases reported in 16 European countries, January 2003 through June 2007*

Variable	MDR TB strains		Unadjusted OR (95% CI)	Adjusted† OR (95% CI)
	No. patients	% Clustered		
Sex				
Male	347	48	1	1
Female	170	49	1.0 (0.7–1.5)	1.1 (0.6–1.9)
Age				
15–44	324	43	1	1
45–64	143	61	2.1 (1.4–3.1)	1.3 (0.7–2.3)
>65	36	53	1.5 (0.7–3.0)	2.8 (0.8–9.4)
Origin				
Baltic states	188	81	**21.9 (10.6–44.9)**	**25.1 (9.9–64.0)**
Other FSU countries‡	99	61	**7.7 (3.7–16.1)**	**8.7 (3.2–23.2)**
Other European countries	72	17	1	1
Africa	71	24	1.6 (0.7–3.6)	1.1 (0.4–3.5)
Site of disease				
Pulmonary	473	50	**2.4 (1.2–4.9)**	0.5 (0.2–1.6)
Extrapulmonary	41	29	1	1
Previous TB				
Yes	163	54	1.0 (0.6–1.4)	1.1 (0.6–1.9)
No	248	55	1	1
XDR TB				
Yes	51	82	**4.1 (1.9–8.7)**	_
No	341	53	1	–

*TB, tuberculosis; MDR, multidrug-resistant; OR, odds ratio; CI, confidence interval; FSU, former Soviet Union; XDR, extensively drug-resistant. **Boldface** indicates significance. †For all other factors in the model except XDR TB. ‡Former Soviet Union countries: Azerbaijan, Belarus, Georgia, Kazakhstan, Kyrgyzstan, Moldova, Russia, Tajikistan, Ukraine, and Uzbekistan.

Univariate analysis showed that origin from the Baltic states or other FSU countries, age category 45–64 years, and pulmonary tuberculosis were significantly associated with clustering. Among these variables, only origin from the Baltic states (adjusted odds ratio [OR] 25.1, confidence interval [CI] 9.9–64.0) or other FSU countries (adjusted OR 8.7, 95% CI 3.2–23.2) remained strongly associated with clustering in the multivariate model. The proportion of XDR TB cases was significantly higher among clustered strains than among unique strains (OR 4.1, 95% CI 1.9–8.7).

### Risk Factors for TB Characterized by Strains of the Beijing Genotype among MDR TB Cases

Analysis to identify Beijing genotype strains was possible for 500 of the 521 MDR TB strains with both epidemiologic and genotyping data (data from Germany and Lithuania were excluded). Of these 500 strains, 284 (57%) were identified as the Beijing genotype and 216 (43%) were non-Beijing genotypes (Figure 1). Univariate analysis demonstrated that origin from the Baltic states or other FSU countries, age categories 45–64 years and >65 years, and pulmonary TB were significantly associated with strains of the Beijing genotype (Table 5). Among these variables, origin from Baltic states and other FSU countries remained strongly associated with Beijing genotype strains in the multivariate analysis. Proportions of clustering and XDR TB were significantly higher among Beijing strains than among other genotype strains, (OR 19.6, 95% CI 12.3–31.2, and OR 5.5, 95% CI 2.1–14.3, respectively). After exclusion of the largest cluster, E0051 (n = 166 cases with epidemiologic data), characterized by a Beijing strain, the association between clustering and the Beijing genotype was 5× less but still quite strong for 334 MDR TB cases with genotyping data (OR 4.5, 95% CI 2.7–7.6).

**Table 5:** Predictors for TB caused by Beijing strains among 506 cases reported in 16 European countries, January 2003 through June 2007*

Variable	MDR TB strains		Unadjusted OR (95% CI)	Adjusted† OR (95% CI)
	No. patients	% Beïjing strains		
Sex				
Male	333	58	1	1
Female	164	55	0.9 (0.6–1.3)	1.1 (0.5–2.4)
Age				
15–44	313	53	1	1
45–64	142	67	**1.7 (1.1–2.6)**	1.0 (0.5–2.1)
>65	30	70	2.5 (1.1–5.5)	2.3 (0.5–10.4)
Origin				
Baltic states	188	92	**154.5 (54.0–442.0)**	**173.8 (45.5–663.6)**
Other FSU countries‡	99	75	**39.7 (14.4–109.5)**	**32.2 (8.6–120.8)**
Other European countries	71	6	1	1
Africa	71	10	1.5 (0.4–4.9)	1.1 (0.2–5.5)
Site of disease				
Pulmonary	455	59	**3.4 (1.7–6.6)**	0.7 (0.1–3.3)
Extrapulmonary	40	30	1	1
Previous TB				
Yes	156	64	1.1 (0.8–1.7)	1.0 (0.5–2.0)
No	245	62	1	1
Clustering				
Yes	250	87%	**19.0 (12.0–30.3)**	–
No	250	26%	1	
XDR TB				
Yes	51	90	**5.5 (2.1–14.3)**	–
No	341	62	1	

*TB, tuberculosis; MDR, multidrug-resistant; OR, odds ratio; CI, confidence interval; FSU, former Soviet Union; XDR, extensively drug-resistant. **Boldface** indicates significance. †For all other factors in the model except XDR TB. ‡Former Soviet Union countries: Azerbaijan, Belarus, Georgia, Kazakhstan, Kyrgyzstan, Moldova, Russia, Tajikistan, Ukraine, and Uzbekistan.

## Discussion

Molecular surveillance of MDR TB cases in Europe showed several large molecular clusters (also including XDR TB cases). The largest cluster (175 cases) was mainly localized in Estonia (information to be completed for Lithuania and Latvia). Origin from the Baltic states and other FSU countries was strongly associated with clustering. The proportion of XDR TB was high among clustered strains. The high proportion of MDR TB case-patients without reported TB history suggests circulation of primary MDR strains in these countries. Three of the 18 European clusters were caused by low-copy strains and may represent false clustering. However, because these clusters consisted of only a few cases, the proportion of European clustering found in this project (43%) is probably only slightly (<3%) overestimated.

A high proportion (55%) of genotyped MDR TB strains belonged to the Beijing genotype family. Strains of the Beijing genotype family were mainly reported for case-patients originating from the Baltic states and other FSU countries, where the prevalence of this genotype is also high in the general population (*12*). The 3 largest clusters were characterized by strains of the Beijing genotype family (84% of the clustered MDR TB cases).

Two hypotheses may explain the occurrence of the large clusters in Europe that were detected in this project. The first hypothesis is direct person-to-person transmission. This hypothesis can be verified by cluster investigation of the cases (by using contact-tracing data if available) in countries with cases reported in clusters. Verifying that the isolates, especially of the largest clusters, represent a single strain is important and can be done by contact investigations and application of an additional typing method such as the newly standardized 24-loci variable number tandem repeat (VNTR) typing (*23*). The second hypothesis is that genetically highly related strains are responsible for most MDR TB cases in Europe and that only a part of the cases in these clusters is associated with direct person-to-person transmission. In both scenarios, conditions could be improved by reinforcing case management (including healthcare access), following up on treatment (especially drug compliance), and increasing social support. Adequate infection control measures should also be ensured in healthcare facilities. The second scenario underlines that more fundamental research (including detailed research on DNA repair, fitness, and transmissibility) is needed to better understand changes in the bacterial population structure of TB, including the drug-susceptible bacterial population and the ongoing evolutionary development of *M. tuberculosis*.

This study was limited by a lack of completeness of the genotyping information and, to a lesser extent, of epidemiologic information. Data completeness varied by country. For some countries (Norway, the United Kingdom, the Netherlands, and Germany), the molecular data reported represent only a fraction of the molecular data available because these countries also used alternative DNA typing methods, which were not included in this project. Other countries have not yet implemented genotyping. Low data completeness in some countries could have affected the proportion of clustered versus unique strains, possibly introducing a bias in the interpretation of the risk factor analysis. However, the proportion of MDR TB cases submitted with both epidemiologic and genotyping information from 16 (68%) countries is promising, considering that IS*6110* RFLP typing is technically demanding. This proportion could probably be improved in the future with the implementation of the recently standardized VNTR typing (*23*).

Another limitation of this study was that the identification of MDR TB clusters was based on genotype clustering. Therefore, person-to-person transmission could not be proven. Ideally, transmission dynamics should be confirmed by conventional contact investigations to link patients to contact persons (*24*). For the main clusters, EuroTB correspondents (from the countries where these clusters have been identified) have received the identification numbers of the clustered MDR TB cases and were able to communicate with colleagues from other countries concerned by the same cluster and exchange information on case histories. However, for the largest MDR TB cluster (E0051), a special field investigation of cases would be necessary and would require international support and coordination. With the limitations of this study, the findings represented here may not be representative of all MDR TB cases in Europe.

MDR TB cases included in clusters should be investigated (including contact-tracing data) in countries where they have been identified to increase knowledge about cross-border transmission of MDR TB and XDR TB and to identify routes of transmission and additional risk factors for these transmission pathways. The outputs of the investigations will enable interventions that prevent further transmission of MDR TB and XDR TB caused by increasing migration between European countries. Standards should be developed to conduct these investigations, including mechanisms to inform concerned national authorities when a molecular cluster is detected and a method for linking epidemiologic and genotyping data at the European level. Delays between diagnosis of MDR TB cases and cluster detection should be reduced to facilitate appropriate actions in the concerned countries and to avoid international transmission of these strains. International collaboration in cluster investigations should be encouraged

Results of this project are preliminary because data are not complete for all countries. Since March 2008, European surveillance of MDR TB has been undertaken by the European Centre for Disease Prevention and Control. A molecular component has been added in this surveillance program. Ideally, this project should be extended to other European Union countries and neighboring countries that could provide genotyping data.
